# Case Report: A case of advanced duodenal adenocarcinoma in complete remission after chemotherapy combined with targeted therapy and radiotherapy

**DOI:** 10.3389/fonc.2022.968110

**Published:** 2022-10-24

**Authors:** Zhengfeng Zhang, Yang Lei, Dazhen Wang, Liu Yang, Changjie Lou

**Affiliations:** Department of Gastroenterology, Harbin Medical University Cancer Hospital, Harbin, China

**Keywords:** case report, duodenal adenocarcinoma, advanced stage, chemotherapy, radiotherapy, targeted therapy

## Abstract

Duodenal adenocarcinoma (DA) is an extremely rare and highly aggressive malignant tumor of the digestive system. Due to the lack of specific clinical characteristics, it is easy to misdiagnosis and miss diagnosis, and the lack of specific consensus and recommendation for treatment, so it often refers to stomach cancer and colorectal cancer. Now, we report a case of a patient with advanced DA who achieved complete remission (CR) after undergoing chemoradiotherapy combined with targeted therapy. The patient was pathologically diagnosed with DA after radical surgery in October 2020, and he failed to undergo adjuvant chemotherapy on time due to the COVID-19 outbreak. The patient found multiple lymph node liver and abdominal metastases 6 months after the operation. Considering the progression of the disease, XELOX regimen (oxaliplatin + capecitabine) chemotherapy was given for 1 cycle. After 1 cycle of treatment, the tumor markers remained elevated; the carcinoembryonic antigen (CEA) was 5.03 ng/ml (0–5 ng/ml), and the carbohydrate antigen 19-9 (CA19-9) was 747.30 U/ml (0–37 U/ml). The patient also developed intolerable capecitabine-related treatment-related adverse events (TRAEs), namely, hand–foot syndrome. For the above reasons, capecitabine was replaced as S-1 at cycle 2, and the chemotherapy regimen became SOX (oxaliplatin + S-1); bevacizumab injection was also added to the SOX regimen, and it was further treated regularly for 7 cycles with the regimen of SOX plus bevacizumab. Liver metastases showed a continuous narrowing trend throughout the treatment period; tumor markers also showed a downward trend. Finally, the patient achieved complete remission (CR) at cycle 7. After completion of chemotherapy, radiotherapy was administered to the resistant metastatic lymph nodes present in the patient’s abdominal cavity for a total of 10 times. However, the patient developed severe bone marrow suppression and obstructive jaundice during the course of radiotherapy and finally failed to complete the radiotherapy plan. Currently, the patient continued maintenance therapy with bevacizumab and S-1 and showed no recurrence or metastasis after review. In this case of advanced DA, we referred to both CRC and gastric cancer in the treatment regimen of the patient. At the same time, targeted drugs and radiotherapy were also added to the basis of chemotherapy, which has no clear consensus recommendation or case for reference in the treatment of advanced DA. Thankfully, the patient’s disease was controlled and remained stable after treatment with this regimen. Therefore, for patients with advanced DA who lack standardized treatment regimens and guidelines, the combination of chemotherapy with targeted therapy and radiotherapy may be one of the effective treatment modalities.

## Introduction

Duodenal adenocarcinomas (DA) represent 0.3%–1% of gastrointestinal malignancies, with an incidence rate of less than 0.5/100,000 (1). However, in terms of occurrence of small intestinal malignancies, DA represents a higher proportion, accounting for approximately 25%–35% (1, 2). Early diagnosis of DA is challenging, as it lacks specific clinical features, so it is prone to missed diagnosis or misdiagnosis as biliary and pancreatic disease and then misses the optimal treatment time and finally develops into advanced DA (3). As a result, the prognosis for the disease is often poor.

Hirashita et al. (4) found that patients with advanced DA had a significantly better prognosis after following the chemotherapy regimen for CRC than the patients who did not receive chemotherapy (P = 0.016). As a result, they believe that advanced DA, despite the poor prognosis, chemotherapy may allow them to achieve a degree of remission after the disease progresses. DA has low morbidity and high mortality rate, which leads to lack of standard diagnosis and treatment guidelines, while chemotherapy often refers to CRC or gastric cancer guidelines. Therefore, some individuals have shifted the strategy of advanced DA therapy to targeted therapy and more specific immunotherapy, but the current effective clinical evidence is still lacking. Here, we discuss the case of advanced DA patients who recover from disease progression after surgery and eventually achieve CR after treatment with chemotherapy plus targeted therapy and radiation.

## Case report

A 53-year-old male patient with no family history or personal history of bowel cancer presented for “intermittent abdominal distention for more than 1 month”. The patient did not experience nausea or vomiting, blood or black stools, abdominal pain or diarrhea, and fever or jaundice during the onset of the disease. Eating, defecation, and body position were not associated with bloating symptoms; urine color deepening was greater than before; and stool was normal.

The routine physical examination: the patient had better mental status, with stable vital signs and no yellowish skin and sclera, no enlarged superficial lymph nodes, and no liver and spleen enlargement affected; both lungs breathing sounds were normal; and no abnormal breathing sounds could be detected. There was no pain, rebound pain, and muscle tension, and bowel sounds can be heard in the whole abdomen, with an average of three to five times/minute. Weight loss was approximately 4–5 kg, and no remaining abnormalities were noted.

After admission, abdominal MRI scan was performed, and the results showed space occupying lesions in the hook region of the pancreatic head region; possibility of malignancy was high; the clinical stage of cT3N1Mx was concomitant, accompanied by bile duct system expansion; and multiple liver metastases may be present. The value of the tumor marker CA19-9 was 94.9 U/ml above the normal reference range (0–37 U/ml), and the CEA and lactate dehydrogenase (LDH) levels were in the normal range. Two ultrasound-guided liver biopsies were performed to clarify the patient’s diagnosis, but these were negative. Finally, a PET/CT examination was performed, and the results showed the following: ampullary space-occupying lesions, possibly ampullary carcinoma; low-density nodules at liver S4 and S8; the tumor possibly showing metastasis; and the paralesional lymph nodes showing radioactive concentration, with high possibility of tumor metastasis. The patient also had low biliary obstruction. Second, we recommend the patient for endoscopic pathology by ultrasound, hoping to undergo surgical treatment after a clear pathologic diagnosis. However, because the patient had undergone two biopsies of liver puncture but had not obtained the pathological tissue, the patient and family members refused to perform endoscopic ultrasonography and hoped to undergo exploratory laparotomy as soon as possible to establish a clear diagnosis. When the opinions of the patients and their families were integrated, the patient did not have obvious organic lesions in the heart, lung, kidney, and other organs, and both liver puncture tests did not show any positive findings in pathological tissue scan. In addition, the patient has developed low biliary obstruction with elevation of tumor landmarks. Consent was obtained after a multidisciplinary consultation to be transferred to surgical therapy.

Downward surgery under epidural composite general anesthesia occurred on 2 October 2020. The surgical name was “exploratory laparotomy + modified pancreaticoduodenectomy”. Surgery was an R0 resection, starting with the resection of the pancreatic head, duodenum, proximal jejunum, distal stomach, common bile duct, and gallbladder, followed by the reconstruction of the three sites: the pancreas and transverse colon, common bile duct and jejunum, and stomach and jejunum. Surgical records showed the following: the surgical incision was located in the right upper quadrant, and the skin was cut about 24 cm through the rectus abdominis incision. Intraoperative exploration showed the following: no abdominal ascites, no implant nodules in the peritoneum, and the right liver surface adhered to the diaphragm, which was released. The imaging showed that the occupation of the liver and the surface of the whole liver were not touched, the volume of the gallbladder increased, and about 200 ml of dark-green bile was drained by decompression. The common bile duct was seen after dissection of the hepatic duodenal ligament, which had dilation, approximately 1.5 cm in diameter. A 1 cm * 1 cm mass was reached in the ampulla region of the inferior common bile duct with a hard texture. Enlargement of hilar lymph nodes, paraciliary choledochal lymph nodes, and common hepatic artery lymph nodes were found. The lesion was found to be resectable after free exploration, so the decision to perform pancreaticoduodenectomy was made. The gallbladder was removed, and a total of 12 parahepatic arteries and common bile duct lymph nodes were removed and submitted for examination. The common bile duct was cut above the gallbladder junction, draining all bile, cutting the common bile duct about 1.5 cm in diameter, and the distal bile duct was ligated. The broken end of the common bile duct was sent to the frozen pathological examination, and no tumor cells were found. The gastric tissue was severed between the gastric body and the antrum, and the pancreas was then exposed. The transverse mesentery was dissected until the inferior margin of the pancreas, revealing the pancreatic head and superior mesenteric arteries, cutting the jejunum at the 10-cm jejunum, distal suture, and proximal suture from the transverse colon mesoic pore pulled back to the right. The pancreatic tissue was cut open after ligating the severed blood vessels of the pancreas, and the posterior pancreatic hook process appeared and the posterior resection mesangium was ligated. Since then, the tissues of the pancreatic head, duodenum, proximal jejunum, distal stomach, and the common bile duct and gallbladder have been completely removed. Reconstruction was then performed. In pancreatic and jejunal anastomosis, after leaving free the distal jejunum, the transverse colon was mentioned above from the transverse colon posterior mesangial fissure, and the anastomosis was made at 5 cm from the jejunal fracture end and the pancreas. In common bile duct and jejunal anastomosis, a longitudinal incision equal to the aperture of the common bile duct was made in the lateral edge of the jejunum from the pancreas and jejunostomy, followed by common bile duct jejunoanastomosis. In gastric jejunum anastomosis, a transverse colonic anterior gastrojejunostomy was performed about 50 cm below the common hepatic jejunal anastomosis. An ampullary carcinoma was intraoperatively diagnosed. However, the postoperative pathology showed a poorly differentiated DA, tumor tissue invaded the base layer of the duodenal intestinal wall involving parenteral adipose tissue, and there is also nerve invasion, pancreatic resection (-), bile duct resection (-), gastric resection (-), duodenal resection (-), and lymph nodes 0/7 in each group. Therefore, this patient was eventually diagnosed with poorly differentiated DA.

After the operation, the patient failed to perform adjuvant chemotherapy on time due to the COVID-19 outbreak. Review at 6 months thereafter revealed an elevation of the tumor marker CA19-9 to 515.5 U/ml; the abdominal MRI found multiple nodules in the liver, low signal on the T1WI sequence, a slightly higher signal in T2WI, some lesions with T2WI, larger nodules located in S6, about 10 mm * 8 mm in size, and a high possibility of metastasis. Combined with the elevation of the patient’s tumor markers, considering the patient’s disease progression, admission to chemotherapy is recommended. The chemotherapy regimen for cycle 1 was the SOX regimen (oxaliplatin+S-1:oxaliplatin: 220 mg per dose, intravenous administration, administered once during a treatment cycle, 21 days is used for one treatment cycle, S-1: each time 60 mg, oral medication, twice daily for 14 consecutive days per treatment cycle). The SOX treatment regimen is guidelines for gastric cancer. Hematological indicators were checked before medication in cycle 2, and it was found that tumor markers continued to rise (CEA: 5.03 ng/ml, CA19-9: 747.30 U/ml). After 1 cycle of chemotherapy, the tumor markers still show a significant upward trend, perhaps indicating that chemotherapy alone cannot effectively control the progression of the disease; after a multidisciplinary consultation, it was agreed that targeted drugs might be added to try them out.

The patient’s primary tumor was located in the duodenum, and postoperative immunohistochemistry was performed: HER-2(1+), VEGF(+), Topo-IIα(+2%), PDGER-α (-), Ki67 (+30%), KRAS (-), NRAS (-), BRAF (+), MLH1 (+50%), PMS2 (+40%), MSH2 (+60%), MSH6 (+40%). The patient developed capecitabine-related adverse drug reactions after 1 cycle of oral capecitabine. The main manifestation is hand–foot syndrome, reaching grade 4. Due to the above reasons, coupled with the patient being not too old and in good physical condition, after consulting with the family members and obtaining their informed consent, capecitabine was replaced with S-1 during cycle 2 of chemotherapy. In addition, we added bevacizumab to conventional chemotherapeutic agents (the dose was 5 mg/kg, administered intravenously, administered once during a treatment cycle, and 21 days was used for one treatment cycle). Then, the patient regularly completed the following 7 cycles of treatment, and the patient tolerated the drug well throughout the treatment period and showed no serious drug-related adverse reactions again.

The patient was reviewed and evaluated on time throughout the treatment period. Evaluation criteria were according to the Response Evaluation Criteria in Solid Tumors version 1.1 (RECIST1.1). The patient was rated as SD at cycle 3 (liver metastatic lesions decreased by 5% compared with before). At cycle 5, liver metastasis decreased by 30% and reached partial response (PR). The metastasis was visually invisible at cycle 7 and was assessed as complete remission (CR). During the entire treatment period from cycle 2 to the end of treatment, the liver metastases continued to decrease (ranging from 5% to 30%), and the tumor markers also decreased (CA19-9 decreased to normal levels from 949.0 U/ml and remained stable). When 8 cycles of treatment were completed, the patient’s liver lesions were visually invisible from the initial 10 mm * 8 mm to the end of treatment.

After the end of chemotherapy, some lymph nodes in the hilar region and peritoneal cavity metastasis could not be significantly reduced compared with those before chemotherapy, which may be related to the tumor heterogeneity and drug tolerance. After a consultation in the radiotherapy department, they recommended abdominal radiotherapy. The original radiotherapy plan was 1.8 Gy each time, a total of 28 times, and a total radiation dose of 50.4 Gy. However, considering that the patient had experienced myelosuppression during chemotherapy and had a platelet decline after the second radiotherapy session (PLT: 70 * 10^9^/l), combined with the patient’s current physical state and disease condition, the radiotherapy schedule was changed to 20 times with a total radiation dose of 36 Gy. After radiotherapy, the abdominal metastatic lymph nodes showed a shrinkage trend, but the patient developed severe myelosuppression (PLT: 30 * 10^9^/l) and jaundice after the 10th radiotherapy and finally failed to complete the radiotherapy plan. Bone marrow suppression seen during the radiotherapy process mainly showed decreased PLT, white blood cell (WBC), and neutrophil (NEU) count. The PLT value decreased to 30 * 10^9^/l ((125–350 * 10^9^/l), and the WBC value had a minimum drop to 1.5 * 10^9^/l (3.5–9.5 * 10^9^/l). The NEU value decreased to 0.8 * 10^9^/l (1.8–6.3 * 10^9^/l).

Up to the third degree according to the myelosuppression scores, the WBC levels were 1.29–1.0 * 10^9^/l, NEU: 0.9–0.5 * 10^9^/l, and PLT: 49–25 * 10^9^/l. During radiotherapy, the patient’s biochemical index was increased. Total bilirubin was 324.3 µmol/l (3.4–21 µmol/l), direct bilirubin was 180.80 µmol/l (0–3.4 µmol/l), indirect bilirubin was 143.50 µmol/l, and total bile acid was 13,000 U/l (5,300–11,300 U/l). The abnormalities of the above test indicators significantly fit with the jaundice symptoms of the patient. Subsequently, the patient underwent interventional therapy with “biliary drainage + biliary stenting”, and the patient’s jaundice symptoms significantly improved 3 days after surgery. However, the patient failed to continue the subsequent radiotherapy for factors such as myelosuppression and secondary infection. At present, the patient has been treated with bevacizumab plus S-1 for half a year, and no disease progression has occurred until June 2022 **(** **Table 1** **).**

**Table 1:** The whole treatment process of the patient ,including date, treatment regimen, cycle and the changes of hematological indicators (CA19-9, CEA, LDH, WBC, NEU, HGB and PLT).

Date	regimen	Cycle	CA19-9	CEA	LDH	WBC	NEU	HGB	PLT
2020/10/2	operation	_	29.49	2.24	126	10.68	8.83	148	132
2021/4/11	XELOX	C1	516.5	4.78	141	5.49	3.52	145	108
2021/5/11	SOX+Bevacizumab	C2	748.3	5.03	210	6.08	3.57	136	103
2021/6/8	SOX+Bevacizumab	C3	180.1	4.21	200	5.01	3.15	127	99
2021/7/7	SOX+Bevacizumab	C4	105.9	3.81	186	4.55	2.2	135	101
2021/8/15	SOX+Bevacizumab	C5	42.1	2.18	150	3.21	1.93	130	98
2021/9/13	SOX+Bevacizumab	C6	21	2.07	188	2.21	1.8	133	89
2021/10/10	SOX+Bevacizumab	C7	15.5	1.87	197	2	1.97	131	78
2021/11/2	SOX+Bevacizumab	C8	10.9	1.8	125	2.2	2.01	139	82
2021/12/20	SOX+Bevacizumab+Radiotherapy	-	8.91	1.78	137	1.5	0.8	122	30
2021/12/31	Biliary stent implantation	-	12.31	2.21	165	2.3	2.12	138	55
2022/1/19-6/21	S-1+Bevacizumab	-	–	–	–	–	–	–	–

**Figure 1:**
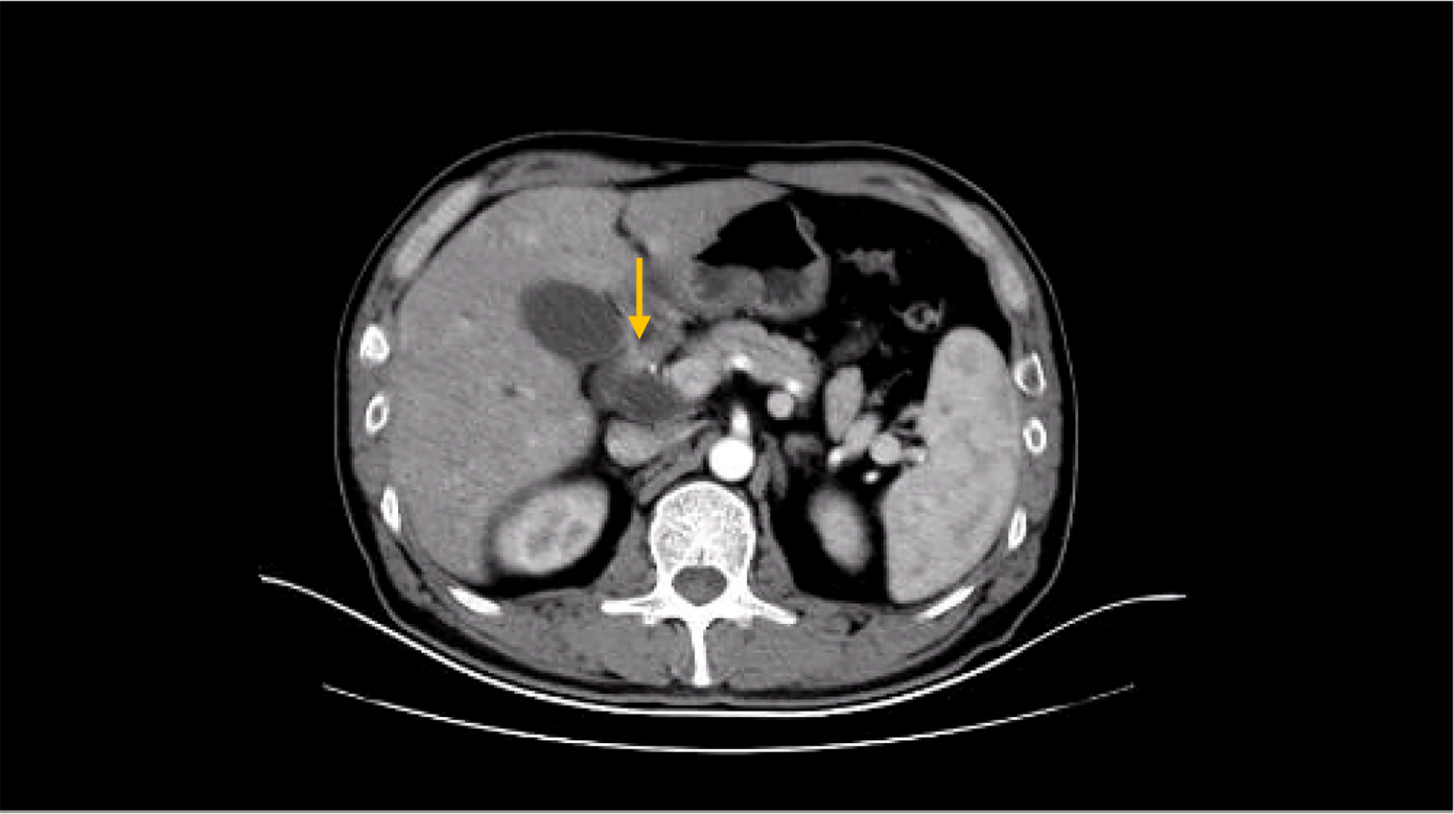
CT before operation (the tumor is located around the head of the pancreas).

**Figure 2:**
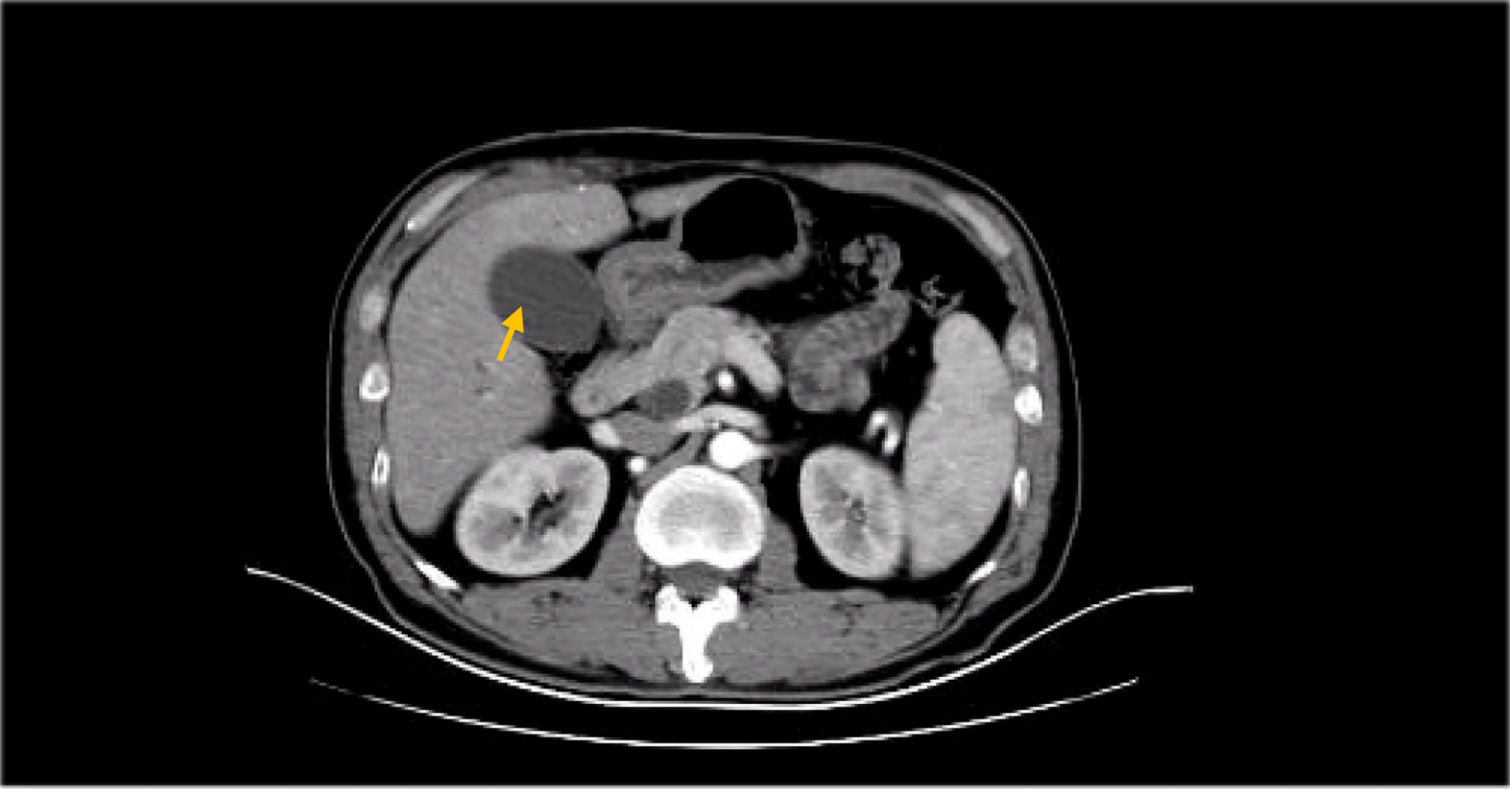
CT (enlargement of gallbladder due to biliary obstruction).

**Figure 3:**
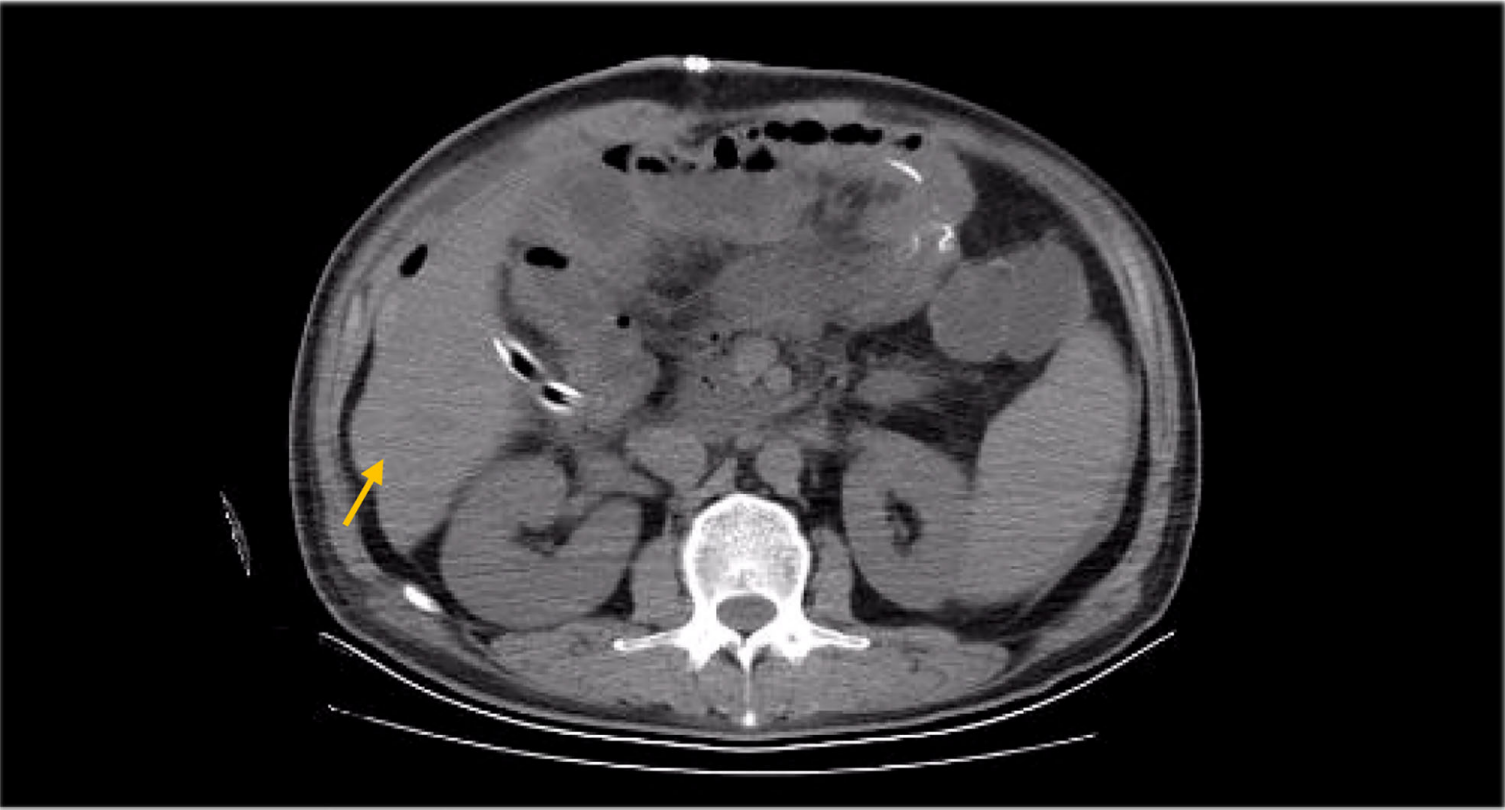
CT findings 1 month after surgery (no liver metastasis).

**Figure 4:**
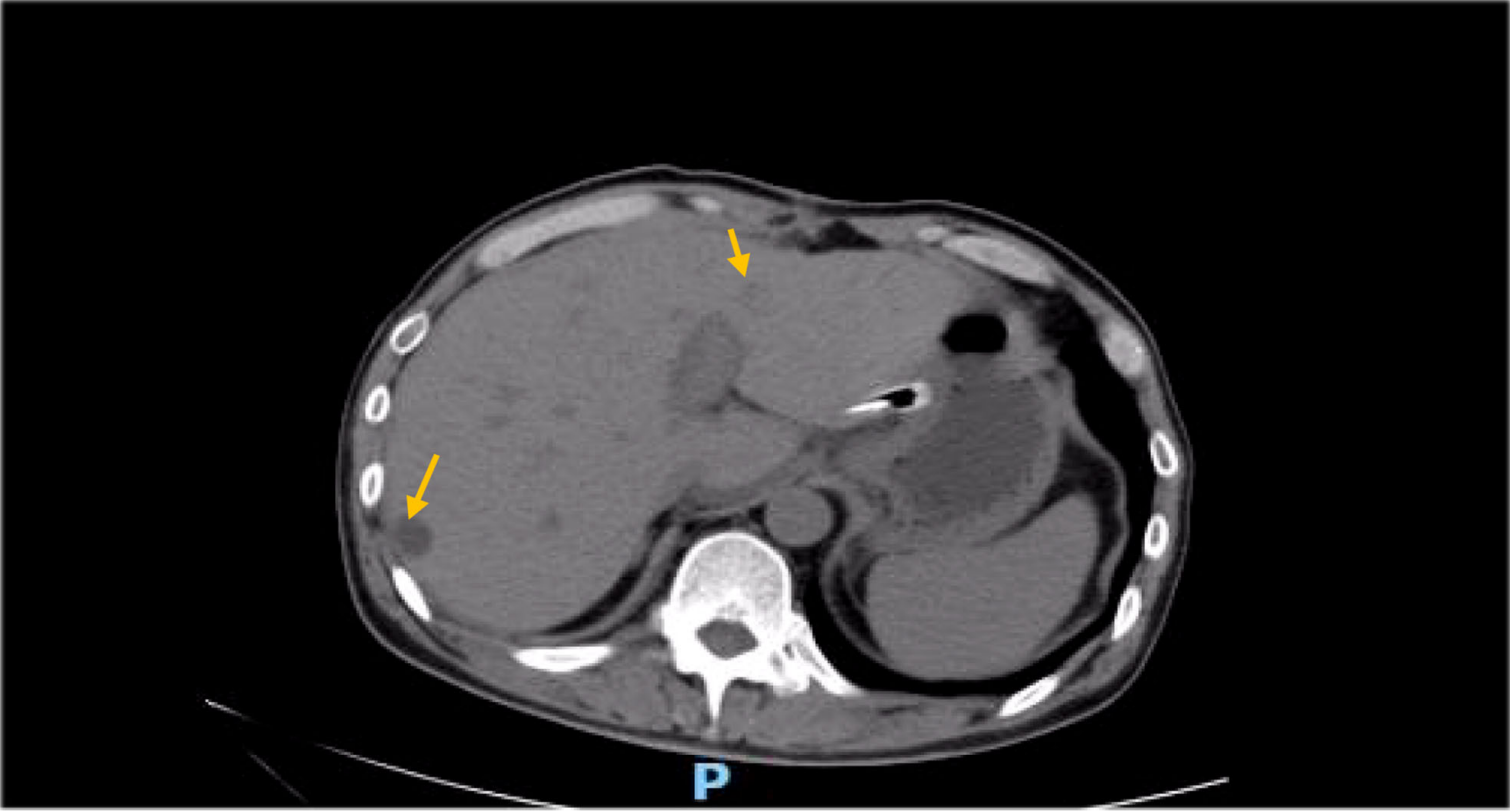
CT (liver metastasis) was performed.

**Figure 5:**
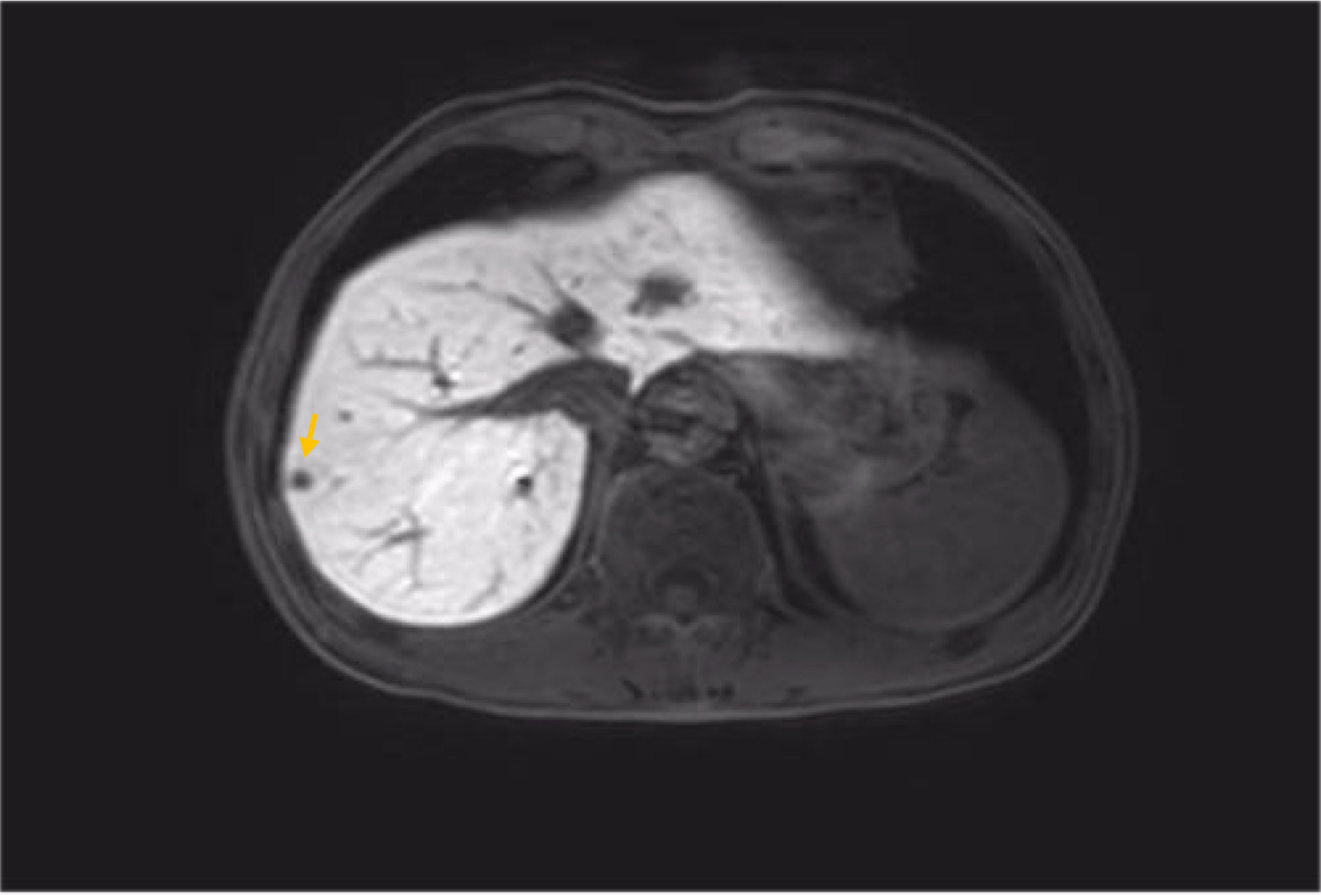
MRI findings 6 months after surgery (liver metastasis).

**Figure 6:**
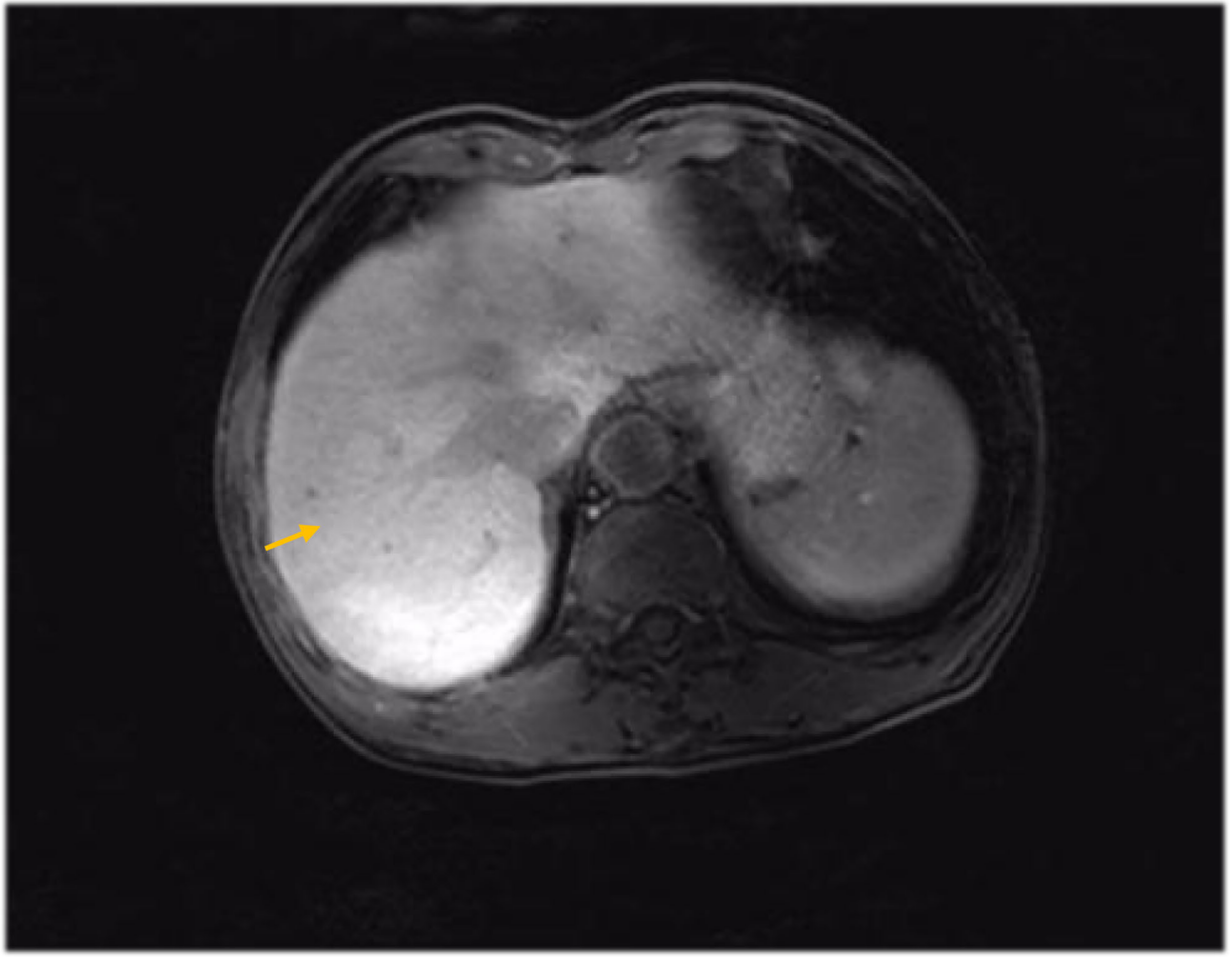
MRI findings during the patient’s last chemotherapy session (liver metastases disappeared).

**Figure 7:**
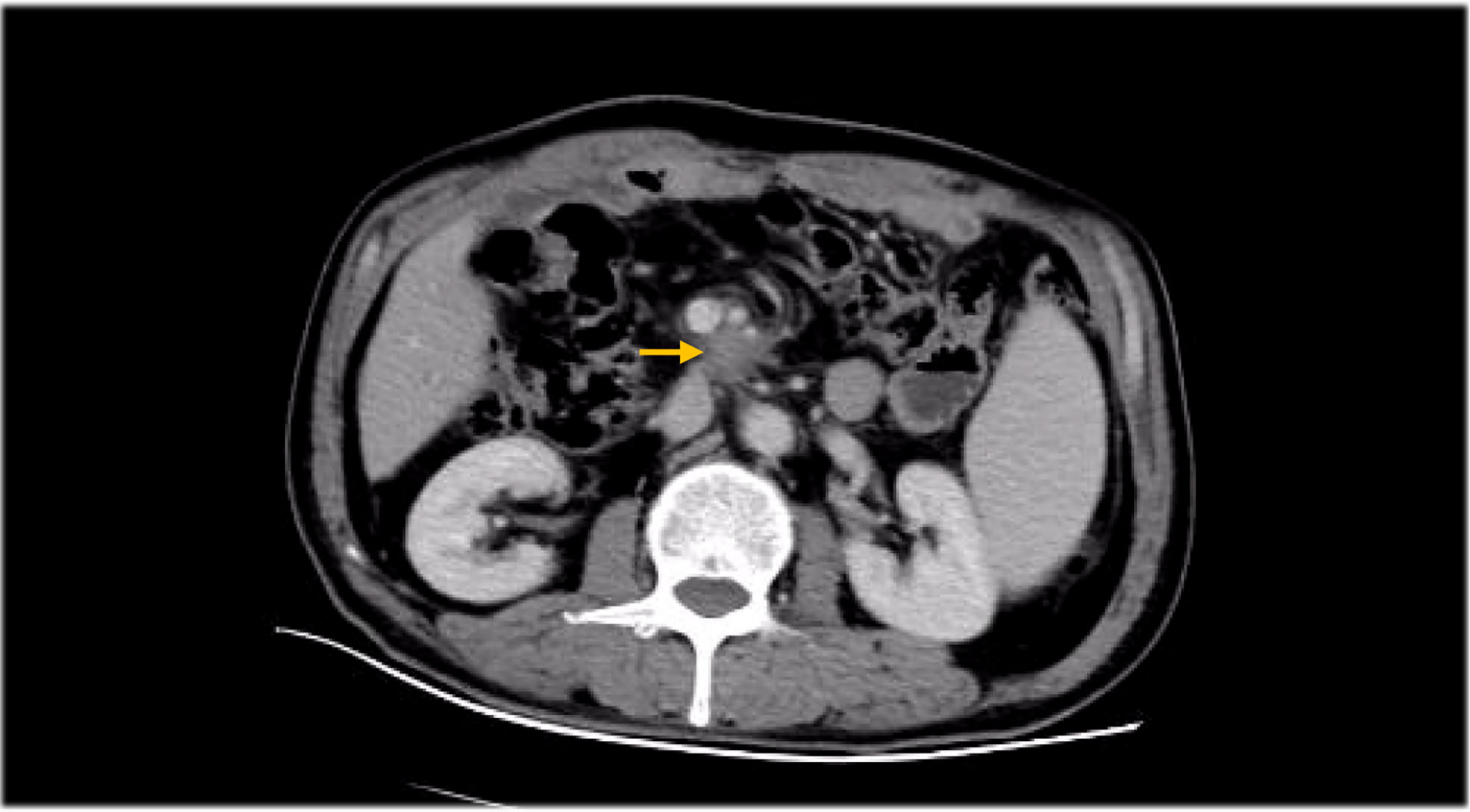
CT Before radiotherapy (abdominal metastatic lymph nodes).

**Figure 8:**
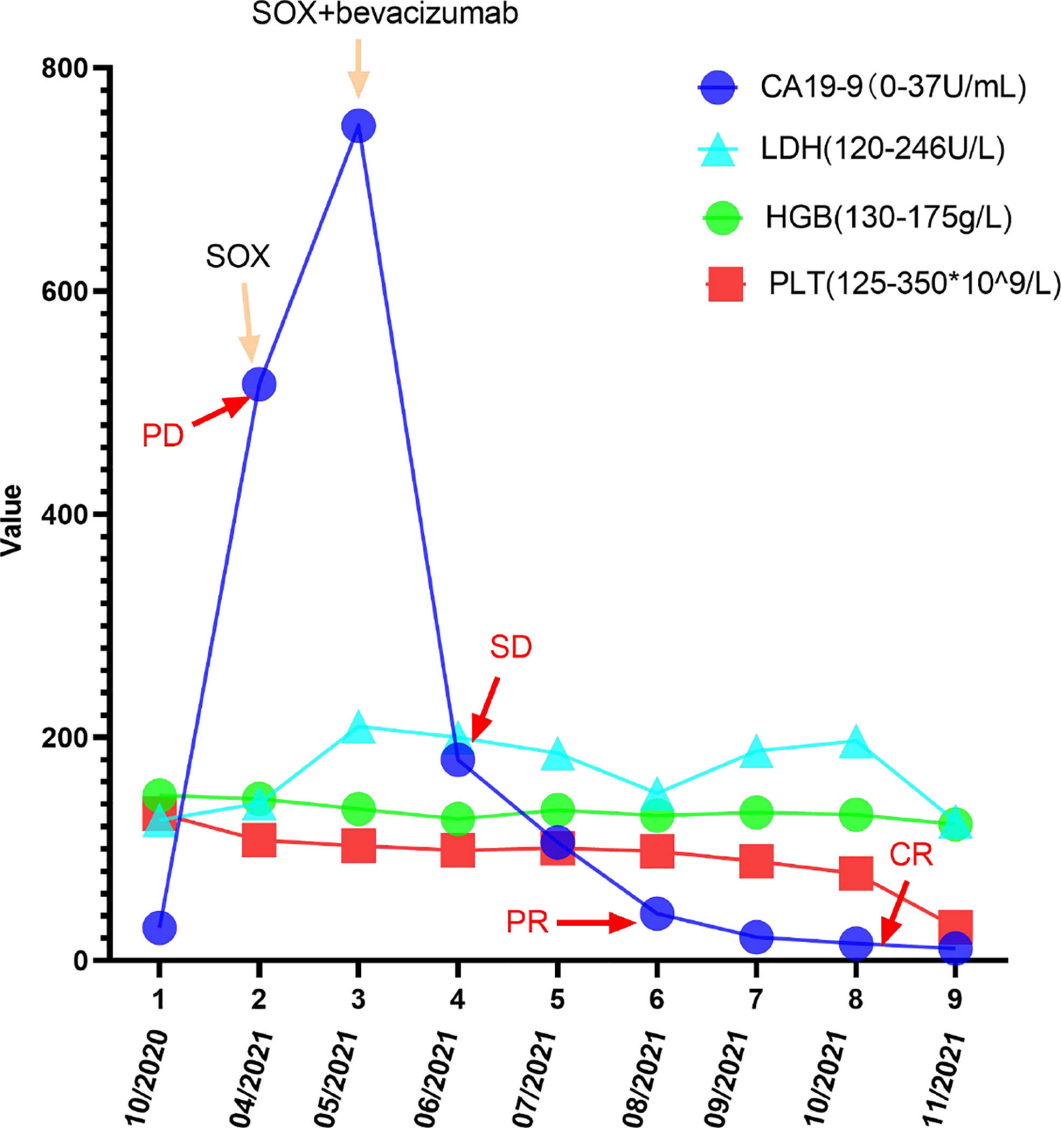
Changes in CA199, LDH, PLT, and HGB levels during the treatment and follow-up. The CA199 LDH, PLT, and HGB levels were measured in the patient’s blood at periodic intervals throughout the clinical course and annotated with date, therapeutic approach, and treatment efficacy.

Besides myelosuppression, drug-related adverse effects exist throughout patient treatment. For example, after the first cycle of capecitabine, the patient developed refractory hand–foot syndrome, mainly showing significant swelling and pain in the palm and foot with severe desquamation and ulcers, as well as local blisters and erythema. Such severe capecitabine-related adverse effects after 1 cycle are rare, as the drug-related adverse effects are usually proportional to the dose. Fortunately, capecitabine was replaced with S-1 in cycle 2, after which the patient gradually resolved. Furthermore, the patient also inevitably developed oxaliplatin-related peripheral sensory neuropathy after 8 cycles of oxaliplatin treatment until the last cycle severity had reached stage 3. This is inevitable, but at this time the patient has successfully completed the treatment and the effect was good **(** **Figures 1**–**8** **).**

## Discussion

The incidence of non-ampullary DA is extremely low, and it has been suggested that the low incidence of DA may be related to the existence of some protective mechanism. For example, the duodenal transport rhythm is fast, which can effectively reduce the exposure of carcinogens; in addition, the presence of intestinal secretions and fluids has a certain protective effect against the relatively sterile duodenum (5). Except for the lower morbidity rate, many DA patients do not show symptoms until the tumor growth is large enough or even develops into an advanced stage, often lacking in specific clinical manifestations, so the early diagnosis is extremely difficult; according to statistics, due to the above reasons, the average delayed diagnosis was about 2 to 15 months, and due to the complex tissue structure of the vicinity, patients are prone to misdiagnosis and missed diagnosis (6, 7).

If the patient can meet the conditions of surgical treatment, the pathological tissue examination after surgical resection can get a more accurate judgment. Although the intraoperative diagnosis is also more credible, many patients’ lesions were located in the ampullary area, there are many surrounding tissues and organs, the structure is more complex, and the lesions are diverse; therefore, the pathological diagnosis is considered to be the most accurate. As reported in the article, the patient was diagnosed with an intraoperative ampullary carcinoma, while the postoperative pathology showed a non-ampullary DA. If the patient is found to have parenchymal organs, such as liver and lung metastases, once examined, the needle biopsy can also be considered for judgment, but it is often prone to negative or false positive results, which is often not accurate enough (7–9). As in the patient in this case, the liver metastasis puncture biopsy was performed twice, but the results were negative, and the intraoperative exploration did not touch the space-occupying lesion shown by the imaging of the whole liver surface. The imaging performance of the primary lesion of the patient in this case was not obvious, even with the possibility of missed diagnosis; in addition, the duodenum belongs to the small intestine, and the location was more special. Either gastroscopy or colonoscopy was difficult to cover, and the examination was mainly to the imaging, supplemented by the sponding hematological indicators.

Although DA is more malignant and progresses rapidly, surgery is still considered the best treatment for DA. A retrospective study was conducted by Juan Manuel Ramia et al. (10) found that the overall 5-year survival rate of all DA patients included in the study was 13% to 50%, and indeed there was some improvement in patients undergoing surgical resection compared with those without surgical treatment, from about 45% to 60%; patients were not surgically treated, the median survival was 7 months, and it was 0%–13% at 5 years (11). Studies have also found the survival status of the patient and the site of the disease. Without lymph node invasion, the survival rate of proximal duodenal tumors is 0% to 25% at 5 years after surgery, while the survival rate of distal tumors is as high as 62% (8, 9, 12, 13). The patient in our case also underwent radical surgical treatment, with surgical R0 resection, but the patient’s disease still progressed rapidly, which may be related to the failure to perform adjuvant chemotherapy timely after surgery. It can be seen that if radical resection can be supplemented with medication on time, it may be more beneficial to the control of the disease.

There is no clear indication and recommendation for the application of postoperative radiotherapy in DA treatment due to the lack of suitable prospective studies. Lim et al. (14) launched a retrospective study of the impact of postoperative adjuvant radiotherapy on survival outcomes in DA patients in 2017; the results found that the later the T stage, especially T4, the larger the tumor size and the higher the proportion of lymph node invasion; they also observed potential survival benefits in postoperative radiotherapy. Regarding the therapeutic effect of radiotherapy in DA, the toxicity of radiotherapy is also an important factor to consider, but due to the rarity of the tumor itself, the adverse effects after radiation are also mostly studies based on small data samples and have not been widely evaluated for (15). In the case we reported, the patient was also supplemented by abdominal radiotherapy after chemotherapy, and we observed a decrease in abdominal metastatic lymph nodes after radiotherapy (due to the COVID-19 outbreak, the patient’s radiotherapy review was not performed in our hospital, so CT imaging images were not provided); however, because the patients later had obvious bone marrow suppression and obstructive jaundice, the radiotherapy plan was unfinished, but we can still think that patients did get some benefit from radiotherapy.

Due to the low incidence of DA, there are also very few prospective studies on the drug treatment of advanced DA, mostly in small and retrospective studies. Zaykowski et al. (16, 17) said in a study of palliative chemotherapy in patients with advanced DA showed that progression-free survival (PFS) in patients receiving advanced chemotherapy extended by 8 months compared with those who received no chemotherapy. In a phase II clinical study of the XELOX regimen for advanced ampullary carcinoma and small bowel carcinoma, conducted by Overman et al. (16, 18), the results showed that the overall response rate (ORR) reached 50%, median time to progression (mTP) was 11.3 months, and median overall survival (mOS) was 20.4 months, indicating that chemotherapy in advanced DA could prolong survival. In addition, Wang et al. (16) once reported that in a patient with advanced DA who received 4 cycles of SOX treatment and 6 cycles of single-agent S-1 maintenance, the PFS reached 14 months, and the toxic effect of the drug administration was fully tolerated during the whole treatment. S-1 is a fluorouracil approved for advanced or metastatic gastric cancer; there is no report or recommendation about its use for DA treatment, but the above evidence shows that S-1 showed good efficacy and safety in patients with advanced DA and can be used as one of the treatment options of advanced DA patients, although further validation is still in clinical practice.

In this patient reported in our article, the first considered regimen was XELOX regimen, but the patient developed refractory capecitabine-related adverse reactions after 1 cycle of medication, so capecitabine was replaced with S-1 in the next cycle of treatment, and the regimen also became SOX. Although the patient was relieved with capecitabine-related adverse effects in the subsequent treatment and had no S-1-related adverse effects, the patient still had other drug-related adverse effects; he still had other drug-related adverse effects, such as oxaliplatin-related peripheral neurotoxicity, even reaching 3° in cycle 8. Oxaliplatin-related or capecitabine-related adverse drug effects are associated with a cumulative dose effect, and in later cases, patients are in the same condition, perhaps to reduce oxaliplatin as appropriate, or try to change the 3-week regimen (oxaliplatin: 135 mg/m^2^ for injection, administered once on 21 days) to a 2-week regimen (oxaliplatin for injection: 85 mg/m^2^, administered once on 14 days). However, although such a treatment plan can reduce a single drug dose and reduce the degree of adverse reactions, the economic and time cost may become another problem. In the treatment of mCRC, the first-line scheme mainly includes oxaliplatin combined with fluorouracil and irinotecan combined with fluorouracil, and with the increase in OS, chemotherapy-related expected toxicity and exposure toxicity will increase, especially oxaliplatin dose-related peripheral neurotoxicity, although after withdrawal there will appear a certain degree of degradation; however, severity can lead to disability, seriously affecting the quality of life of patients (19, 20). Compared with oxaliplatin, irinotecan is generally well tolerated and its clinically relevant cumulative toxicity is less (19). Therefore, if patients develop disease progression again, they can consider irinotecan combined with fluorouracil to be considered as a treatment regimen, hoping to reduce the treatment toxicity of patients and increase the quality of life on the basis of prolonging the patient’s survival.

Hirashita et al. (2) conducted a retrospective study of 25 patients with non-ampullary duodenal adenocarcinoma, mainly investigating the prognostic factors affecting patients with non-ampullary duodenal adenocarcinoma, and the benefit of chemotherapy in relapsed patients referring to the mCRC regimen; they found that the serum CA19-9 level, primary tumor size, depth of tumor invasion, lymph node metastasis, TNM stage, presence of lymphatic metastasis, vascular and nerve infiltration, and other factors were all important risk factors for recurrence; in addition, the relapsed patients receiving chemotherapy according to the mCRC regimen had a significantly better prognosis than those patients without chemotherapy (P = 0.016). The case we reported also had neurological invasion in this patient, which may also be related to the patient’s disease progression.

The retrospective study conducted by Sakae et al. (21) also recommended high LDH and symptoms at diagnosis as new independent prognostic factors for OS. Unfortunately, the patients in our case showed no increase in LDH throughout the course; therefore, it is unknown whether LDH can be used as an independent influencing factor for DA prognosis. In addition, it has been reported that the survival rate of patients without lymph node metastasis was significantly higher than the rate in patients with lymph node metastasis (2, 22, 23). Therefore, it has been suggested to use lymph node metastasis as an important prognostic factor affecting the overall survival of DA patients, with a sensitivity of about 80% (2, 5, 24). We reported in this case that patients have reached R0 resection during surgery, but after the recurrence of liver metastasis they also had multiple abdominal lymph node metastasis, and the abdominal lymph nodes still failed to reach CR after chemotherapy. Abdominal metastasis of lymph nodes may affect the survival of patients; this is also the reason for the continued radiotherapy after the end of chemotherapy.

A case of advanced DA was reported by Kanehira et al. (25); the patient underwent chemotherapy after a laparoscopic gastrojejunostomy, using the chemotherapy regimen of S-1, plus cisplatin, then the abdominal CT after chemotherapy was reviewed; the results showed tumor shrinkage and that the enlarged abdominal lymph nodes almost disappeared, after which the patient continued with S-1 maintenance chemotherapy for 1 year without recurrence. Although combination chemotherapy with S-1 and cisplatin is the standard treatment for advanced gastric cancer in Japan, there is no standard regimen for duodenal cancer (26). Wang et al. (16) once reported a case of a patient with advanced duodenal adenocarcinoma achieving CR after chemotherapy with the SOX regimen, which is very similar to the case in our text. Onkendi et al. (27) reported that patients were treated with leucovorin plus 5-FU plus irinotecan (FOLFIRI) or oxaliplatin (FOLFOX); disease remission rates in DA can be improved to some extent, but CR is still rare. In short, between the low incidence of duodenal cancer, large clinical research is difficult, and the standard treatment strategy is still a long way to go; however, reference to either gastric cancer or bowel cancer treatment is needed according to the patient-specific situation, to a certain extent, and the doctor’s clinical experience and reference to typical successful cases are also needed.

For patients with advanced DA, the effect of chemotherapy on the long-term prognosis is relatively limited, so targeted therapy may be a potential direction to explore. After all, molecular targeted therapy has shown good results in multiple solid tumors. Human epidermal growth factor receptor-2 (Her-2), which is currently widely studied in adenocarcinoma, mainly promotes tumor invasion and metastasis by activating signaling pathways including Ras/MAPK and PI3K/Akt (28). At present, the overexpression of Her-2 and the amplification of the ERBB2 gene have been widely used in the treatment of breast cancer, gastric cancer, and colorectal cancer and have become a therapeutic target for prolonging patient survival, but its therapeutic potential in DA lacks clear evidence and sufficient data to support (28–30). Hamad et al. (31) added anti-Her-2 targeted drugs to the treatment of ERBB2 amplified patients with advanced DA, and combined with FOLFOX for neoadjuvant therapy, in the efficacy analysis, the patient showed significant tumor decline and no metastasis; moreover, no residual invasive adenocarcinoma was found in the postoperative pathological analysis, which was basically consistent with the previous neoadjuvant therapy response.

Gulhati et al. (32) conducted a single-center phase II clinical study of a bevacizumab combined with XELOX regimen in adenocarcinoma of advanced small bowel or an ampullary method to evaluate the benefit of combining bevacizumab with XELOX; there were 23 patients with small bowel adenocarcinoma, and the primary study endpoint was a 6-month PFS, with a 6-month PFS probability of 68% after combined bevacizumab, an ORR of 48.3%, an mPFS of 8.7 months, and a mOS of 12.9 months. Therefore, the XELOX regimen combined with bevacizumab is considered a feasible combination in the treatment of advanced DA, and this study is also the first prospective clinical study to evaluate the feasibility of targeted therapy in small bowel cancer.

In recent years, there have been several retrospective studies showing that patients with metastatic colon cancer with the primary tumor located on the right side (ileocecal to splenic area) have a significantly worse prognosis than on the left side (splenic to rectum). For patients with no mutations in the RAS gene, there was a clear relation between the efficacy of the anti-EGFR (cetuximab) and the tumor site and no significant association with site efficacy of anti-VEGFR (bevacizumab) (1). Moreover, a comparative chemotherapy combined with bevacizumab or cetuximab retrospective subgroup analysis shows the following: in the left CRC, cetuximab objective efficiency and overall survival better than bevacizumab, and in the right CRC, cetuximab but with some advantages in objective efficiency, although with overall survival than bevacizumab (19). Moreover, there is evidence that up to 96% of patients with small intestinal adenocarcinoma express VEGF-A, and the expression level of VEGF-A mRNA in ampullary duodenal carcinoma is significantly higher than that of adjacent normal intestinal mucosa (33, 34). This is one of the reasons why we chose bevacizumab as a targeted agent.

In this case report, the patient only underwent routine immunohistochemistry, without large-sample genetic testing, and more genetic mutations were unclear, but the addition of bevacizumab for vascular endothelial growth factor receptor (VEGFR) also showed good efficacy. Therefore, we can think that bevacizumab combined with chemotherapy can benefit patients with advanced DA to some extent. Moreover, some alterations in the target genes can indeed be the targets for the treatment of patients, especially for the tumors with a high malignant degree and some rare diseases, such as DA.

Except for targeted therapy, the rapid research progress of immunotherapy is also an aspect worthy of attention. After all, research on immune checkpoint inhibitors (ICIs) in recent years has indeed made relatively good achievements in many solid tumors, including esophageal cancer and CRC; for example, advanced esophageal squamous cell carcinoma has included ICIs in the first line. In DA patients, patients who also have microsatellite instability high (MSI-H) or deficiency of mismatch repair (dMMR) may also be the target population for ICI treatment. Studies have been found that 21% of patients in DA can have dMMR, which is significantly higher than 5% of CRC. Therefore, despite the low incidence of DA, the blockade against immune checkpoints is likely to be a significant opportunity for (35, 36). The patient reported in the case is currently relatively stable after chemoradiotherapy combined with targeted therapy. If the patient advances again in the later stage, then immunotherapy must also be a thing to be considered.

Early detection, early diagnosis, and early treatment must be the fundamental measures to improve the survival of DA patients. DA is often difficult due to its unique adjacent results and histological characteristics, so how to effectively screen people at high risk of DA is extremely urgent. Studies have shown that familial polyposis, adenomatous disease, Crohn’s disease, hereditary non-polyposis colorectal cancer, and Peutz–Jeghers syndrome (6, 8, 37). In addition, duodenal adenoma is also a recognized risk factor for the onset of DA, and its clinicopathological classification is called Spigelman classification, in which stage IV has the highest risk degree, and about 35% of patients with Spigelman stage IV develop (24, 38). Some research data also show that the duodenal polyp size and the emergence of high dysplasia are components of Spigelman stage IV, suggested as an important predictor of cancer risk; only the detailed reference criteria on this staging system or evaluation of treatment relevance has not yet been developed, which we need to continue to explore and study (4, 24).

In conclusion, due to the low incidence and extreme malignancy of DA, it is unrealistic to carry out large-scale clinical randomized controlled experiments, and research on the diagnosis and treatment of the disease is also greatly challenged. The patient we reported in this case, although already with advanced DA, was well controlled after treatment with SOX combined with bevacizumab. Therefore, in the diagnosis and treatment of DA, early detection is the best, but if unfortunately entering the advanced stage, actively receiving chemotherapy or using it in combination with targeted therapy, radiotherapy, and even immunotherapy may also greatly delay the progress of the disease. In summary, there is still a clear lack of guidelines and consensus for the diagnosis and treatment of advanced DA, and there is still a long way to go for the treatment of DA patients.

## Data availability statement

The original contributions presented in the study are included in the article/supplementary material. Further inquiries can be directed to the corresponding author.

## Ethics statement

The manuscript complies with the requirements of medical ethics and ethics, with the informed consent of the patient.

## Author contributions

All authors listed have made a substantial, direct, and intellectual contribution to the work, and approved it for publication.

## Funding

Harbin Medical University Cancer Hospital Haiyan Scientific research fund (JJZD2020-03).

## Conflict of interest

The authors declare that the research was conducted in the absence of any commercial or financial relationships that could be construed as a potential conflict of interest.

## Publisher’s note

All claims expressed in this article are solely those of the authors and do not necessarily represent those of their affiliated organizations, or those of the publisher, the editors and the reviewers. Any product that may be evaluated in this article, or claim that may be made by its manufacturer, is not guaranteed or endorsed by the publisher.
